# Combined Iron Chelator and Antioxidant Exerted Greater Efficacy on Cardioprotection Than Monotherapy in Iron-Overloaded Rats

**DOI:** 10.1371/journal.pone.0159414

**Published:** 2016-07-18

**Authors:** Suwakon Wongjaikam, Sirinart Kumfu, Juthamas Khamseekaew, Jirapas Sripetchwandee, Somdet Srichairatanakool, Suthat Fucharoen, Siriporn C. Chattipakorn, Nipon Chattipakorn

**Affiliations:** 1 Cardiac Electrophysiology Research and Training Center, Faculty of Medicine, Chiang Mai University, Chiang Mai, Thailand; 2 Cardiac Electrophysiology Unit, Department of Physiology, Faculty of Medicine, Chiang Mai University, Chiang Mai, Thailand; 3 Center of Excellence in Cardiac Electrophysiology Research, Chiang Mai University, Chiang Mai, Thailand; 4 Department of Oral Biology and Diagnostic Sciences, Faculty of Dentistry, Chiang Mai University, Chiang Mai, Thailand; 5 Department of Biochemistry, Faculty of Medicine, Chiang Mai University, Chiang Mai, Thailand; 6 Thalassemia Research Center, Institute of Molecular Bioscience, Mahidol University, Nakhon Pathom, Thailand; Max-Delbrück Center for Molecular Medicine (MDC), GERMANY

## Abstract

**Background:**

Iron chelators are used to treat iron overload cardiomyopathy patients. However, a direct comparison of the benefits of three common iron chelators (deferoxamine (DFO), deferiprone (DFP) and deferasirox (DFX)) or an antioxidant (N-acetyl cysteine (NAC)) with a combined DFP and NAC treatments on left ventricular (LV) function with iron overload has not been investigated.

**Methods and Findings:**

Male Wistar rats were fed with either a normal diet or a high iron diet (HFe group) for 4 months. After 2 months, the HFe-fed rats were divided into 6 groups to receive either: a vehicle, DFO (25 mg/kg/day), DFP (75 mg/kg/day), DFX (20 mg/kg/day), NAC (100 mg/kg/day) or the combined DFP and NAC for 2 months. Our results demonstrated that HFe rats had increased plasma non-transferrin bound iron (NTBI), malondialdehyde (MDA), cardiac iron and MDA levels and cardiac mitochondrial dysfunction, leading to LV dysfunction. Although DFO, DFP, DFX or NAC improved these parameters, leading to improved LV function, the combined DFP and NAC therapy caused greater improvement, leading to more extensively improved LV function.

**Conclusions:**

The combined DFP and NAC treatment had greater efficacy than monotherapy in cardioprotection through the reduction of cardiac iron deposition and improved cardiac mitochondrial function in iron-overloaded rats.

## Introduction

The iron overload condition is often seen in transfusion dependent thalassemia (TDT) patients due to repeated blood transfusions, and also in hereditary hemochromatosis patients due to increased dietary iron absorption into the duodenal enterocytes [1–5]. Iron overload leads to increased free iron (labile iron) in the plasma which is called non-transferrin bound iron (NTBI) [6, 7]. The accumulation of excess plasma NTBI leads to an increase in the entry of free iron into cells and this accumulation causes the dysfunction of many cellular organelles and also to direct tissue damage, especially in the heart [6–8]. Excess cardiac iron accumulation can cause iron overload cardiomyopathy which is a common cause of death in these patients [1–5]. It has been shown that free iron enters into the heart mainly through L-type calcium channels (LTCC) [9, 10], and T-type calcium channels (TTCC) [11, 12] under conditions of iron overload. Cardiac iron toxicity occurs when there is too much labile iron in the cell and this free iron reacts with superoxide (O_2_•-) and hydrogen peroxide (H_2_O_2_) via Haber-Weiss and Fenton’s reactions to produce the highly toxic hydroxyl radical (•OH). The production of this free radical leads to an increase in the levels of reactive oxygen species (ROS) [13, 14] and cardiac oxidative stress, leading to damages of cardiac cells which results in cardiac dysfunction and heart failure [14, 15]. Moreover, increase ROS production under iron overload condition led to plasma membrane lipid peroxidation which resulted in the increase of cytotoxic aldehydes, especially malondialdehyde (MDA) via Haber-Weiss and Fenton’s reactions [16]. MDA is very toxic to cells and highly increased in cardiac iron overload, leading to cardiac dysfunction in a murine model [16]. Previous studies also found that MDA was increased both in plasma and tissue of iron-overloaded rats [17–19].

The production of O_2_•- and H_2_O_2_ is derived largely from mitochondrial activity particularly in the heart [20, 21]. Iron interacts with O_2_•- and H_2_O_2_, which are supplied by the mitochondrial electron transport [22]. Iron-catalyzed oxidants cause mitochondrial DNA damage and mitochondrial dysfunction, leading to a loss of respiratory capacity and cardiac dysfunction [23]. Further evidence of this is documented in a previous study which demonstrated that iron overload caused increased cardiac ROS production which led to cardiac mitochondrial depolarization and swelling in isolated cardiac mitochondria of wild type and β-thalassemic mice [24].

Currently, three common iron chelators including deferoxamine (DFO), deferiprone (DFP) and deferasirox (DFX) are used to treat and prevent cardiac iron deposition and cardiac dysfunction in iron overload cardiomyopathy [25–33]. Nonetheless, direct comparisons between the therapeutic effects of DFO, DFP and DFX on the heart under conditions of iron overload have not yet been investigated. Moreover, growing evidence showed that N-acetyl cysteine (NAC) is a potent antioxidant and a precursor of an antioxidant glutathione [34] which can scavenge free radicals such as H_2_O_2_ and •OH in cells [35]. Under an iron overloaded condition, NAC provided protective effects on brain dysfunction in iron-overloaded rats [18], and it may also reduce ROS levels and DNA damage in β-thalassemia patients [36]. Interestingly, combined DFP plus NAC therapy has been shown to provide synergistically therapeutic benefits in improving and restoring brain function which was impaired by iron toxicity [18]. However, the protective effects of NAC alone or combined DFP plus NAC on cardiac dysfunction caused by the iron overload condition have not yet been investigated. This study aimed to test the hypothesis that pharmacological interventions with DFO, DFP, DFX, NAC or combined DFP plus NAC can attenuate cardiac iron concentration, diminish cardiac oxidative stress, and improve left ventricular (LV) function and cardiac mitochondrial function in iron-overloaded rats, and that a combination of a chelator with NAC therapy can synergistically provide beneficial effects for these conditions.

## Materials and Methods

### Animal preparation

All animal studies were approved by the Institutional Animal Care and Use Committee (IACUC) at the Faculty of Medicine, Chiang Mai University (Permit number: 21/2557). Forty-two adult male Wistar rats, body weight 180–200 g, were obtained from the National Laboratory Animal Center, Mahidol University, Bangkok, Thailand. Animals were housed in an animal holding room under controlled conditions at 23 ± 2°C, 50 ± 10% humidity and 12 h light/dark cycles. The animals were acclimatized for a week prior to the experiment and received drinking water *ad libitum* throughout the entire experiment.

### High iron diet preparation

A chow diet containing 0.2% of ferrocene (C_10_H_10_Fe; Sigma-Aldrich, Co., St. Louis, USA) (w/w) was prepared as described previously [18]. In brief, one kilogram of a chow diet was grinded and 2 g of ferrocene were added with mixing well. Then, 1.2 L of deionized water was added in the iron-loading diet and mixed well again. After that, iron loading diet was molded and baked at 80°C for 24 hours in a hot air oven.

### Experimental protocols

**Fig 1:**
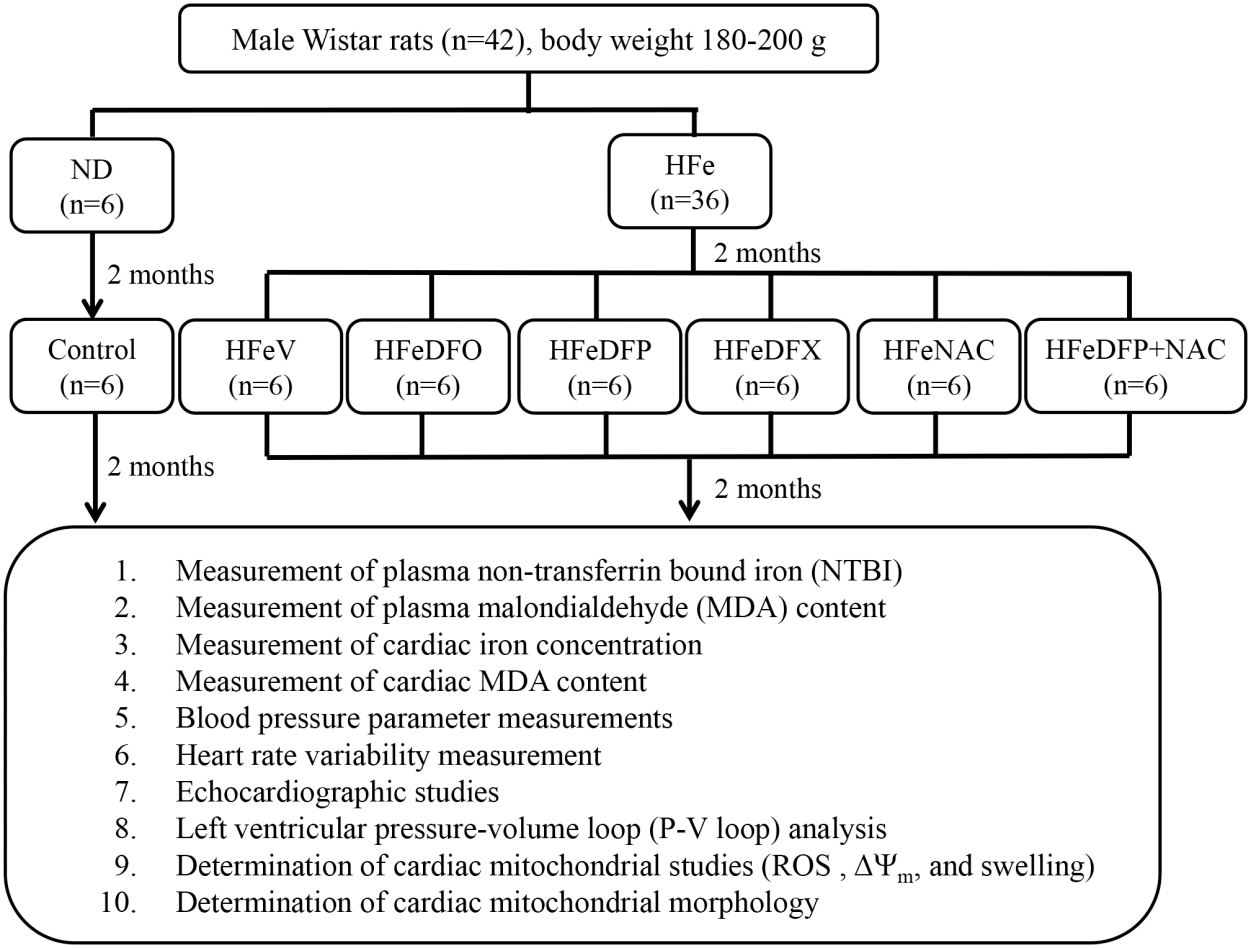
The summary of the experimental protocol. ND = normal diet group; HFe = high iron diet group; V = vehicle; DFO = deferoxamine; DFP = deferiprone; DFX = deferasirox; NAC = N-acetyl cysteine; NTBI = non-transferrin bound iron; MDA = malondialdehyde; ROS = reactive oxygen species; ΔΨ_m_ = membrane potential change.

Adult male Wistar rats (180–200 g) were divided into 2 groups to receive either a normal diet (a chow diet, ND) (n = 6), or high iron diet (0.2% ferrocene w/w, HFe) (n = 36) for 4 months. Two months into this protocol, ND-fed rats received a vehicle (normal saline solution, NSS) (control group) once a day via either subcutaneous injection (n = 3) or gavage feeding (n = 3) and continued with their normal diet for 2 months. HFe-fed rats were divided into 6 groups (n = 6/group) including 1) HFe given NSS (HFeV) once a day via either subcutaneous injection (n = 3) or gavage feeding (n = 3), 2) deferoxamine (DFO; Desferal^®^, Novartis Pharma Stein AG, Stein, Switzerland), (HFeDFO) 25 mg/kg/ day via subcutaneous injection, 3) deferiprone (DFP; Ferriprox^®^, Apotex Inc.,Toronto, Ontario, Canada), (HFeDFP) 75 mg/kg/day, 4) deferasirox (DFX; Exjade^®^, ICL670, Novartis Pharma Stein AG, Stein, Switzerland), (HFeDFX) 20 mg/kg/day, 5) N-acetyl cysteine (NAC, Sigma-Aldrich, Co., St. Louis, USA), (HFeNAC) 100 mg/kg/day, or 6) combined DFP 75 mg/kg/day plus NAC 100 mg/kg/day (HFeDFP+NAC) via gavage feeding for 2 months, and all of these groups were continuously fed with the high iron diet. Although DFO could remove cardiac iron in patients with cardiac iron loading, the route of DFO administration is a subcutaneous or an intravenous injection which causes sufferring in patients who are treated with this iron chelator. In addition, DFP showed to be more effective than DFO in removing excess cardiac iron store in TDT patients [27, 37]. DFX is an expensive oral iron chelator compared to the cost of DFP. Moreover, previous studies demonstrated that DFP was more effective than DFX in reducing severe cardiac iron loading in TDT patients [37, 38]. Therefore, DFP was chosen to combine with NAC in this study. At the end of the experiment (4 months after the start of the diets) heart rate variability (HRV), echocardiography and left ventricular pressure-volume (P-V) loop analysis were determined in all groups. Rats were deeply anesthetized by intraperitoneal injections of Zolitil (ZolazepamTiletamine) 50 mg/kg combined with Xylazine 3 mg/kg [39], and then P-V loop analysis was performed for 20 minutes. After P-V loop analysis, the blood was collected to measure plasma non-transferrin bound iron (NTBI) and plasma malondialdehyde (MDA) levels. Then, the deeply anesthetized rats were sacrificed by rapidly removal of the heart. The heart was used to determine cardiac iron concentration, cardiac MDA content and the study of cardiac mitochondria in all groups. The summary of the experimental protocol is shown in Fig 1.

### Measurement of plasma non-transferrin bound iron (NTBI) concentration

To determine the presence of an iron overload condition, plasma NTBI level was measured using nitrilotriacetic acid disodium salt (NTA) chelation/flow cytometry [40, 41] with some modifications. Plasma was incubated with NTA solution (at a final concentration of 80 mM) pH 7.0 for 30 minutes at room temperature to allow Fe^3+^-(NTA)_2_ complexes to form. Then, the Fe^3+^-(NTA)_2_ complexes were separated from the plasma proteins by spinning the plasma mixture through a membrane filter (NanoSep^®^ 30-kDa cut off, polysulfone type; Pall Life Sciences, Ann Arbor, MI, USA). The concentration of Fe^3+^-(NTA)_2_ complexes represented NTBI in the ultrafiltrate. Ferric nitrate was used as a standard and was prepared at a final concentration of 0–10 μM. Standard or Plasma- Fe^3+^-(NTA)_2_ was added with chelatable fluorescent beads (5X10^6^ beads/mL) (1:1), followed by 180 μL of 50 mM MOPS buffer pH 7.0 and inserted into a 96 well microplate. Then, it was mixed and shaken in the dark for 2 hours. The NTBI (via Fe^3+^-(NTA)_2_ complexes) concentrations were measured by flow cytometry (Guava EasyCyte HT, Merck Millipore, Germany) and were calculated from a standard curve using GraphPad Prism software.

### Measurement of malondialdehyde (MDA) concentration

To determine plasma and cardiac MDA concentrations to assess the oxidative stress state of the heart samples were measured using the high-performance liquid chromatography (HPLC) method [42]. Heart tissues were homogenized in a phosphate buffer solution (pH 2.8), containing with butylated hydroxytoluene (BHT) which is an antioxidant in the homogenized buffer, 1:10 (w /v) on ice. Homogenated heart tissue or plasma was mixed with 10% trichloroacetic acid containing BHT (50 ppm), heated at 90°C for 30 minutes, and then cooled for 2 minutes, followed by centrifugation at 6,000 rpm for 10 minutes. After that, the supernatant was mixed with 0.44M H_3_PO_4_ and 0.6% thiobarbituric acid solutions and then incubated at 90°C for 30 minutes. Plasma or cardiac MDA content was determined by absorbance detected at 532 nm by the HPLC system, and was calculated directly from the standard curve.

### Measurement of cardiac iron concentration

Cardiac iron concentration was determined by using a colorimetric assay for measuring a non-heme iron concentration in heart tissues [11, 43]. Heart tissues were homogenized in deionized water 1:10 (w /v) on ice. Homogenated heart tissue was precipitated in protein precipitated solution (1:1) and heated at 95°C for 1 hour. After that, the tubes were cooled for 2 minutes, vortex mixed, and then centrifuged at 8,200 g for 10 minutes. The supernatant was mixed in a ferrozine solution (1:1) and was incubated at room temperature for 30 minutes. After incubation, the absorbance was measured at 562 nm, and the cardiac iron concentration was calculated directly from the iron standard curve.

### Blood pressure parameter measurements

Blood pressure measurements from the tail vein of animals were performed by using a non-invasive blood pressure system (Kent Scientific Corporation, Wyoming, USA) [44, 45]. Animals were preheated under an infrared warming pad for at least 5 minutes to dilate their tail veins and acclimatize to the holder. Then, volume pressure recording and occlusion cuffs were attached to the tail. The blood pressure parameters, including systolic pressure, diastolic pressure and mean arterial pressure were recorded by taking an average of 20 consecutive measurements at a steady state.

### Heart rate variability (HRV) measurement

Cardiac autonomic nervous activity was evaluated using spectral analysis of R-R interval variability. The electrocardiogram (ECG) was recorded for 15 minutes in each rat using the Power Lab system (Power Lab 4/25T, AD instrument) with chart 5.0 software. Data from the ECG recording was analyzed by using the MATLAB program [46]. A power spectrum of R-R interval variability was obtained by use of the Fast Fourier Transform (FFT) algorithm. Three major oscillatory components are detectable using this system: 1) the high frequency component (HF; about 0.6–3 Hz which varies with respiration), which is associated with parasympathetic activity, 2) the low frequency component (LF; about 0.2–0.6 Hz) which is associated with sympathetic and parasympathetic activity, and 3) the very low frequency component (VLF; the power below 0.2 Hz). To minimize the effect of changes in total power on the LF and HF components, LF and HF were expressed as normalized units (LFnu and HFnu) by dividing it by the total power minus VLF. The LF/HF ratio is considered an index of cardiac sympathetic/parasympathetic tone balance [47, 48]. An increase in LF/HF ratio indicates a cardiac sympathovagal imbalance [49, 50].

### Echocardiographic studies

Left ventricular function was determined using an echocardiography (GE Vivid I) [50]. Rats were lightly inducted and maintained with 2% isoflurane with oxygen (2 L/min) inhalation. Their chest areas were shaved and the rats were stabilized for 1–2 minutes in the supine position prior to starting the protocol. The probe was gently placed on the chest and moved to enable the collection of data along the short and long axes of the heart. Signals from M-mode echocardiography at the level of the papillary muscles were recorded. Parameters from the echocardiograph, including left ventricular internal diameter end systole (LVIDs) and end diastole (LVIDd), were recorded. Fractional shortening was calculated by using the following formula: %FS = (LVIDd−LVIDs) x 100 /LVIDd [50].

### Left ventricular pressure-volume (P-V) loop analysis

Left ventricular P-V loop analysis was carried out to assess left ventricular function using a pressure-volume conductance catheter system [39, 51]. Rats were anesthetized by intraperitoneal injections of Zolitil (ZolazepamTiletamine) 50 mg/kg combined with Xylazine 3 mg/kg [39]. Then, the neck was opened with a ventral midline incision and ventilated with room air from a positive pressure ventilator by a Harvard rodent ventilator model 683 (Harvard Apparatus, Massachusetts, USA). It was started immediately with room air using a volume of 200–250 μL and ventilator rate of 70–110 breaths/min to maintain PCO_2_, PO_2_, and pH parameters of the physiological condition. The right carotid artery was canulated with a pressure-conductance catheter (Scisense, Ontario, Canada) [39, 51] which was used for measuring left ventricular pressure and volume for 20 minutes. Heart rate, left ventricular end-systolic pressure (LVESP) and end-diastolic pressure (LVEDP), maximum and minimum pressures (Pmax and Pmin), cardiac output and stroke volume were measured in each rat.

### Cardiac mitochondrial isolation

After P-V loop analysis, heart tissues were rapidly removed, minced and then homogenized in mitochondrial isolation buffer (MIB), pH 7.2 on ice. Then, homogenated heart tissue was centrifuged at 800 g, 4°C for 5 minutes. The supernatant was collected and centrifuged at 8,800 g, 4°C for 5 minutes. Then, mitochondrial pellets were re-suspended in MIB and centrifuged at 8,800 g, 4°C for 5 minutes. Finally, the mitochondrial pellet was re-suspended in a respiration buffer [52]. Protein concentration was determined using the Bicinchoninic Acid assay [53].

### Determination of cardiac mitochondrial reactive oxygen species (ROS) production

The fluorescent dye dichlorohydro-fluorescein diacetate (DCFDA) was used to determine the level of ROS production in cardiac mitochondria [39, 52]. Cardiac mitochondria (0.4 mg/ mL) were incubated at 25°C with DCFDA for 20 minutes. DCFDA can pass through the mitochondrial membrane, and is oxidized by ROS in the mitochondria into DCF (a fluorescent form) [39, 52]. Fluorescence was excited at 485 nm and the emission fluorescence was recorded at 530 nm using a fluorescence microplate reader (Bio-Tek Instruments, Inc. Winooski, Vermont USA). The ROS level was expressed as arbitrary units of fluorescence intensity of DCF.

### Determination of cardiac mitochondrial membrane potential change (ΔΨ_m_)

Mitochondrial membrane potential change was determined by using the dye 5,50,6,60-tetrachloro-1,10,3,30-tetraethylbenzimidazolcarbocyanine iodide (JC-1) and was determined as fluorescence intensity using a fluorescent microplate reader [39, 52]. JC-1 is characterized as a cation and remains in the mitochondrial matrix in a monomeric (green fluorescence) form. In addition, it can interact with anions in the mitochondrial matrix to form an aggregate (red fluorescence) form [39, 52]. JC-1 monomer and aggregate fluorescence were excited at the same wavelength of 485 nm. The emission fluorescence of JC-1 monomer and aggregate was detected at 530 and 590 nm, respectively. Cardiac mitochondrial depolarization was assessed by a decrease in the red/green fluorescence intensity ratio.

### Determination of cardiac mitochondrial swelling

Mitochondrial swelling was studied by using the isolated-mitochondrial suspension [39, 52]. The change in the absorbance of the mitochondrial suspension was measured at 540 nm using a microplate reader (Synergy HT, Bio Tek, Winooski, Vermont, USA). Cardiac mitochondrial swelling was evaluated by a decrease in the absorbance of the mitochondrial suspension.

Cardiac mitochondrial morphology was also determined by using transmission electron microscopy [39, 52]. Cardiac mitochondrial isolations were fixed overnight in 2.5% glutaraldehyde in 0.1 M cacodylate buffer, pH 7.4 at 4°C. Then, they were post-fixed with 1% cacodylate-buffered osmium tetroxide at room temperature for 2 hours. After that, the pellets were dehydrated in a graded series of ethanols and embedded in Epon-Araldite. Ultrathin sections were cut using a diamond knife and placed on copper grids. Finally, they were stained with uranyl acetate and lead citrate, and cardiac mitochondrial morphology was observed using a transmission electron microscope.

### Statistical analysis

Data in each experiment were expressed as mean ± standard error of mean (SEM). The data were processed using SPSS (Statistical Package for Social Sciences, Chicago, IL, USA) release 17.0 for Windows. Student’s *t* test and One-way ANOVA analyses were performed to test for differences between groups. NTBI concentrations were analyzed using a non-parametric Kruskal-Wallis H, followed by a Mann-Whitney U test. *P* values <0.05 were considered as indicating a statistically significant difference between groups.

## Results

### The effects of the pharmacological interventions on plasma NTBI and plasma MDA levels

**Fig 2:**
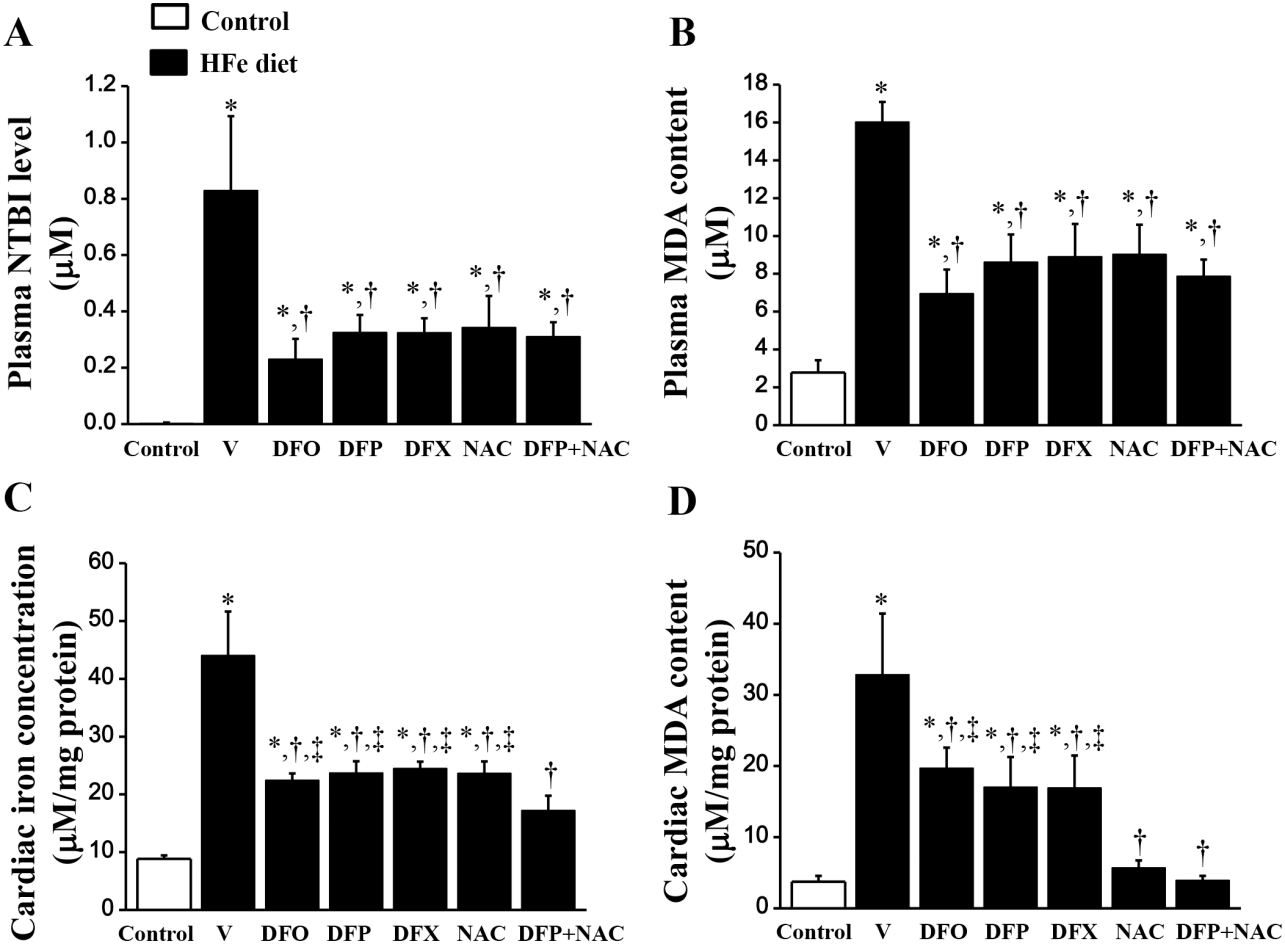
Combined DFP+NAC treatment could decrease plasma NTBI and MDA levels as well as restore normal cardiac iron and MDA content in iron-overloaded rats. The effects of the pharmacological interventions on: (A) plasma non-transferrin bound iron (NTBI) level; (B) plasma malondialdehyde (MDA) content; (C) cardiac iron concentration and (D) cardiac MDA content in iron-overloaded rats. *P < 0.05 vs. control, †*P* < 0.05 vs. HFe, ‡*P* < 0.05 vs. HFeDFP+NAC.

Plasma NTBI was significantly increased in the HFe-fed rats, while levels of plasma NTBI could not be detected in the control group (Fig 2A), indicating that an iron overload condition occurred in the HFe-fed rats. After 2 months of treatment with DFO, DFP, DFX, NAC and combined DFP plus NAC, plasma NTBI level was significantly reduced in the HFe-fed rats (Fig 2A). Plasma MDA level was significantly increased in the HFe-fed rats when compared with the control group (Fig 2B). All of the pharmacological interventions showed a correlation with a significantly decreased plasma MDA level in the HFe-fed rats (Fig 2B). This finding suggests that iron overload increased oxidative stress as shown by increased levels of a lipid peroxidation product (MDA) in the plasma of HFe-fed rats. Moreover, these pharmacological interventions also provided similar efficacy in lowering plasma NTBI and MDA levels.

### The effects of the pharmacological interventions on cardiac iron concentration and cardiac MDA content

Iron concentration was significantly increased in the heart tissue of the HFe-fed rats when compared with the control group (Fig 2C), whereas DFO, DFP, DFX or NAC treatment showed similar efficacy in attenuating cardiac iron concentration. However, only combined DFP plus NAC decreased cardiac iron concentration to a normal level (i.e. the same as the control group) (Fig 2C). Consistently, cardiac MDA content was significantly increased in the HFe-fed rats when compared with the control group (Fig 2D). DFO, DFP and DFX showed the same efficacy in reducing cardiac MDA content, while NAC and combined DFP plus NAC decreased cardiac MDA content to a normal level the same as the control group (Fig 2D). These results suggest that the combination of DFP and NAC treatment led to the restoration of both cardiac iron concentration and cardiac MDA content in iron-overloaded rats.

### The effects of the pharmacological interventions on blood pressure parameters

There was no significant difference in the blood pressure parameters including systolic pressure, diastolic pressure, and mean arterial pressure between the HFe-fed rats and the control group (Table 1). Similarly, systolic pressure, diastolic pressure and mean arterial pressure were not significantly different in any of the pharmacological intervention groups when compared with vehicle-treated HFe-fed rats (Table 1).

**Table 1:** The effects of the pharmacological interventions on blood pressure parameters in iron-overloaded rats.

Groups	Systolic Pressure(mmHg)	Diastolic Pressure(mmHg)	Mean Arterial Pressure(mmHg)
Control	129±3	88±3	101±3
HFeV	125±5	93±4	103±4
HFeDFO	126±4	92±5	103±4
HFeDFP	127±2	92±4	103±3
HFeDFX	127±5	85±5	99±5
HFeNAC	125±1	87±2	100±1
HFeDFP+NAC	128±1	90±5	102±3

Data are shown as mean ±SE. HFe; high iron diet, V; vehicle, DFO; deferoxamine, DFP; deferiprone, DFX; deferasirox, NAC; N-acetyl cysteine, SE; standard error.

### The effects of the iron overload condition and the pharmacological interventions on heart rate variability (HRV)

**Fig 3:**
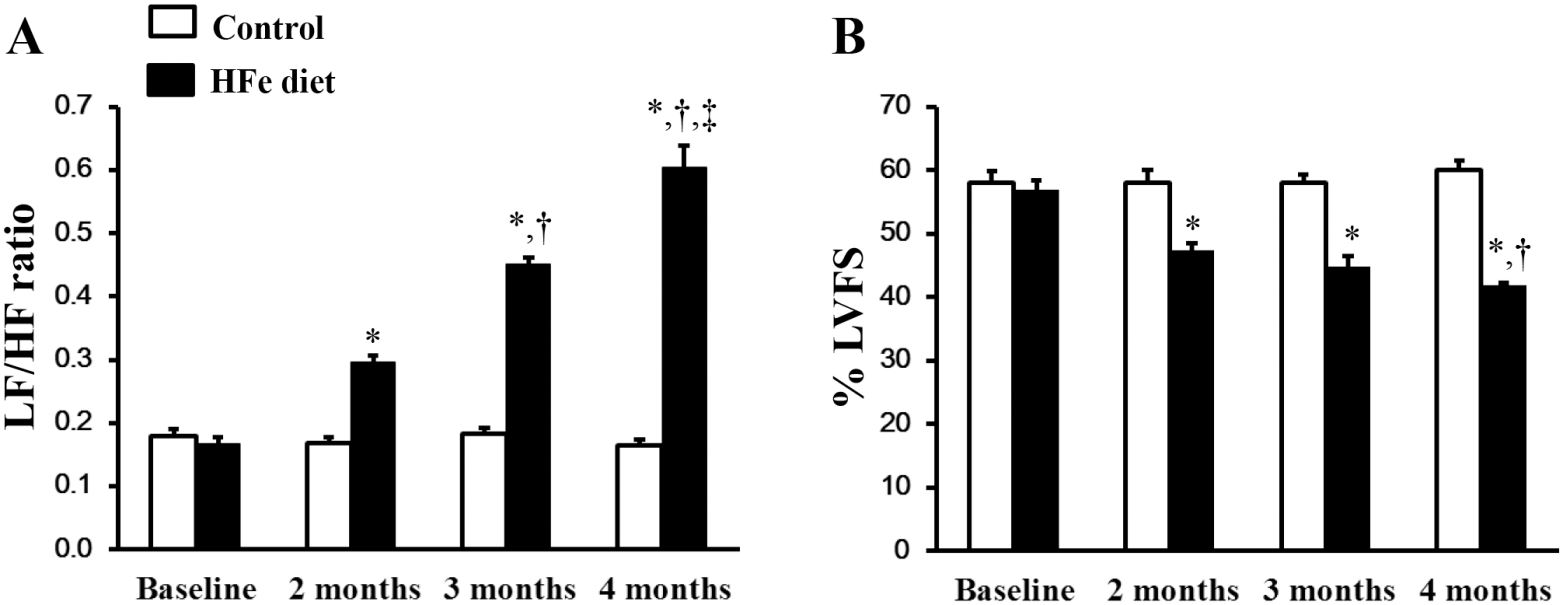
Chronic iron overloaded impaired heart rate variability and decreased cardiac function in iron-overloaded rats. The effects of the iron overload condition on: (A) heart rate variability at baseline, 2, 3 and 4 months, and (B) the percentage of left ventricular fractional shortening (%LVFS) measured using echocardiography at baseline, 2, 3 and 4 months in iron-overloaded rats. *P < 0.05 vs. control, †*P* < 0.05 vs. HFe diet for 2 months, ‡*P* < 0.05 vs. HFe diet for 3 months.

**Fig 4:**
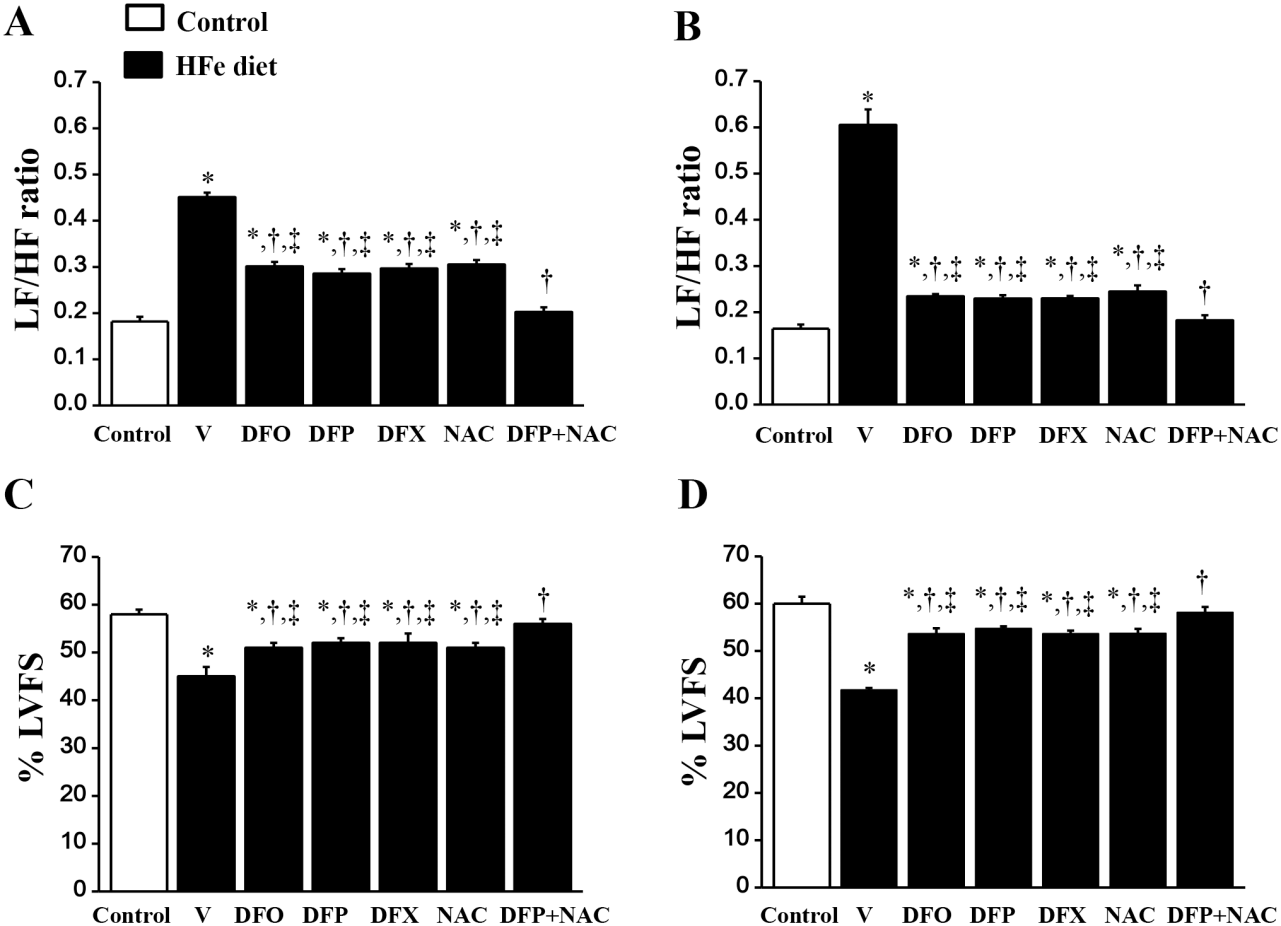
Combined DFP+NAC treatment could restore heart rate variability and cardiac function in iron-overloaded rats. The effects of the pharmacological interventions on heart rate variability at 3 months (A), and 4 months (B), and the percentage of left ventricular fractional shortening (%LVFS) measured using echocardiography at 3 months (C), and 4 months (D) in iron-overloaded rats. *P < 0.05 vs. control, †*P* < 0.05 vs. HFe, ‡*P* < 0.05 vs. HFeDFP+NAC.

At baseline, the LF/HF ratio did not differ between HFe-fed rats and the control group (Fig 3A). After iron was administered for 2, 3 and 4 months, the LF/HF ratio was significantly increased in the HFe-fed rats in a time-dependent manner when compared with the control group. These findings indicate a cardiac sympathovagal imbalance in the rats with iron-overload after 2 months of HFe feeding, and it increased successively at 3 months and 4 months, respectively (Fig 3A). DFO, DFP, DFX or NAC treatments had similar effects in attenuating the LF/HF ratio in the HFe-fed rats thus indicating improved cardiac sympathovagal balance after 1 month (at 3 months) and 2 months (at 4 months) of treatment, respectively (Fig 4A and 4B). However, only the combined DFP plus NAC treatment led to significant attenuation of the LF/HF ratio in the iron-overloaded rats to a control value after 1 month of treatment (Fig 4A), and continued to restore HRV after treatment with the combination of an iron chelator and an antioxidant for 2 months (Fig 4B). This result suggests that the combination of DFP and NAC treatment can significantly improve and restore the HRV in rats with iron-overload rats.

### The effects of the iron overload condition and the pharmacological interventions on cardiac function

From echocardiographic data, the percentage of left ventricular fractional shortening (%LVFS) did not differ at baseline between the HFe-fed rats and the control group (Fig 3B). However, beginning at 2 months after HFe feeding, %LVFS was significantly decreased in the HFe-fed rats when compared with the control group, indicating left ventricular dysfunction in the iron-overloaded rats, and it continued to decrease at 3 and 4 months after HFe feeding (Fig 3B). DFO, DFP, DFX or NAC therapy could improve %LVEF after 1 month of treatment (Fig 4C), and these treatments did continue to improve cardiac function after 2 months of treatment (Fig 4D). Although DFO, DFP, DFX or NAC monotherapy significantly increased %LVFS, only the combination of DFP plus NAC increased %LVFS in the HFe-fed rats to the same level as shown in the control group after 1 month of treatment (Fig 4C). These results remained the same at 2 months of treatment (Fig 4D). These findings suggest that the combination of DFP and NAC treatment can significantly improve and restore LV function in iron-overloaded rats, and provide more robust effects than monotherapy with either an iron chelator or an antioxidant alone.

The P-V loop data demonstrated that HFe-fed rats had significantly decreased LVESP, Pmax, cardiac output and stroke volume when compared with the control group, indicating left ventricular dysfunction in these rats (Table 2). Treatments with DFO, DFP, DFX and NAC significantly improved LVESP, Pmax, cardiac output as well as stroke volume in the HFe-fed rats (Table 2). Only the combined DFP plus NAC treatment led to the restoration of LVESP, Pmax and cardiac output in iron-overloaded rats (Table 2). These results suggest that combined DFP plus NAC treatment exerted greater efficacy than monotherapy in improving LV function in iron-overloaded rats.

**Table 2:** The effects of the pharmacological interventions on hemodynamic parameters in iron-overloaded rats.

Hemodynamic parameters	Control	HFeV	HFeDFO	HFeDFP	HFeDFX	HFeNAC	HFeDFP+NAC
Heart rate (beats / min)	253±2	233±9	240±11	237±7	255±18	235±17	253±12
LVESP (mmHg)	141±5	110±5*	126±4*,†	124±5*,†	124±6*,†	125±3*,†	132±1†
LVEDP (mmHg)	24±1	20±3	23±2	25±2	22±3	26±3	25±2
Pmax (mmHg)	144±4	115±4*	129±5*,†	128±5*,†	129±6*,†	128±3*,†	135±2†
Pmin (mmHg)	20±0.2	15±3	17±2	21±2	18±3	21±3	21±1
Cardiac output (mL/min)	132±4	91±4*	111±3*,†	111±6*,†	112±8*,†	110±5*,†	118±8†
Stroke volume (μL)	517±13	396±10*	434±9*,†	447±12*,†	434±13*,†	436±4*,†	459±7*,†

LVESP, LVEDP, left-ventricular end-systolic and end-diastolic pressure; Pmax, Pmin, maximum and minimum pressure.

*P < 0.05 vs. control,

^†^*P* < 0.05 vs. HFe.

### The effects of the pharmacological interventions on cardiac mitochondrial function in rats with iron- overload

**Fig 5:**
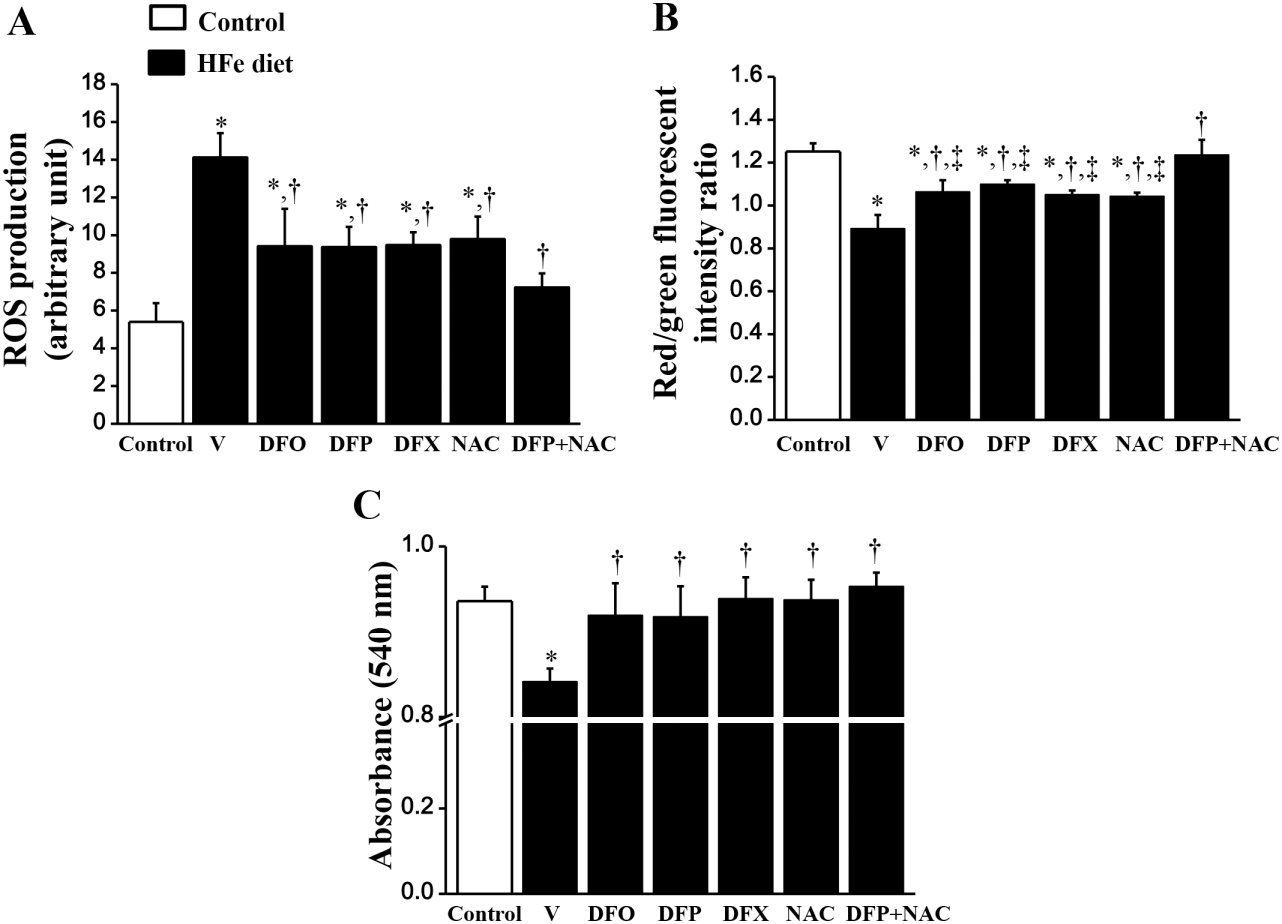
Combined DFP+NAC treatment could restore cardiac mitochondrial function in iron-overloaded rats. The effects of the pharmacological interventions on: (A) cardiac mitochondrial reactive oxygen species (ROS) production; (B) cardiac mitochondrial membrane potential change and (C) cardiac mitochondrial swelling in iron-overloaded rats. *P < 0.05 vs. control, †*P* < 0.05 vs. HFe, ‡*P* < 0.05 vs. HFeDFP+NAC.

**Fig 6:**
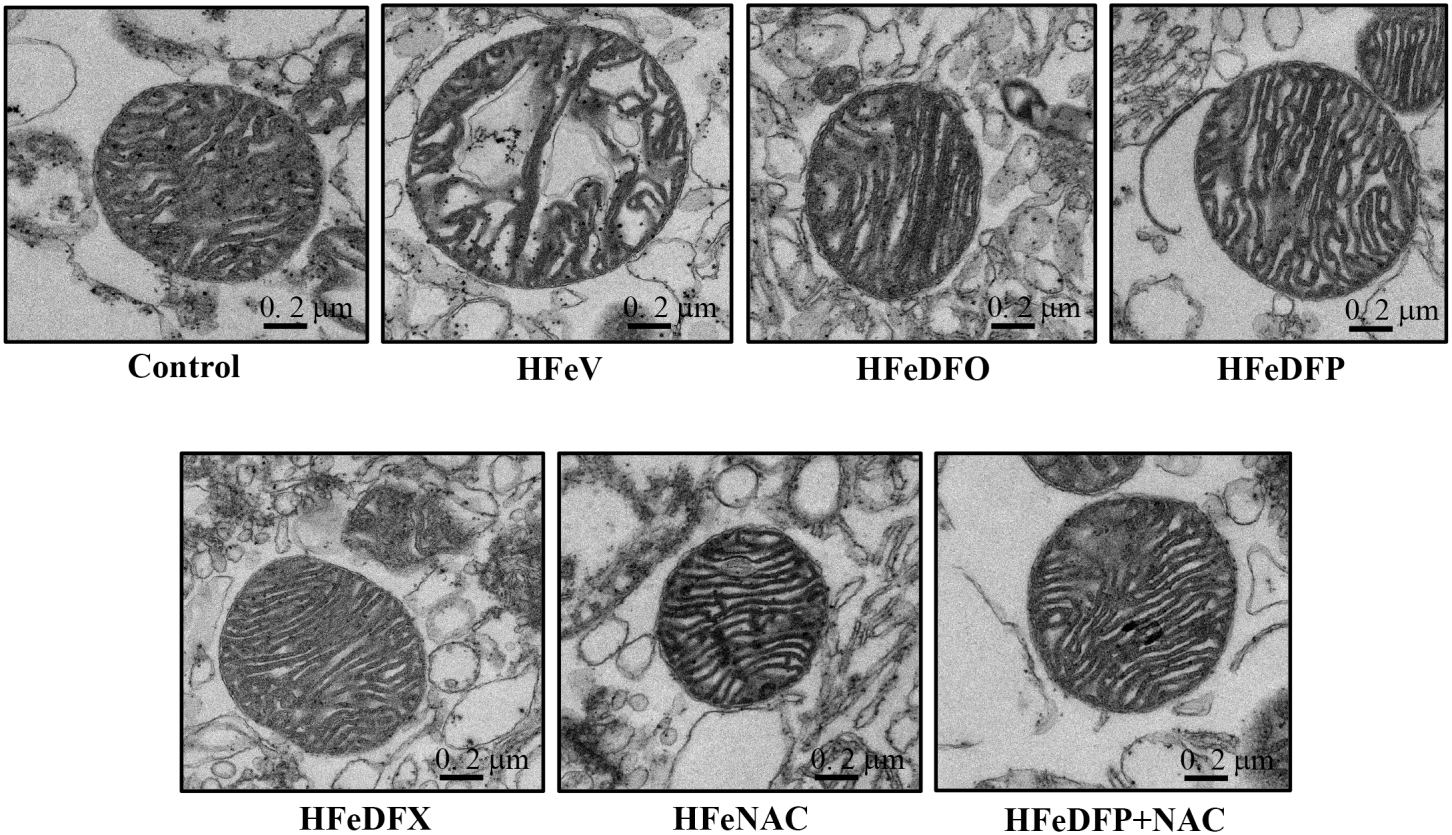
Electron micrographs of cardiac mitochondria from control, HFeV, HFeDFO, HFeDFP, HFeDFX, HFeNAC and HFeDFP+NAC groups. Iron-overloaded rats led to dysmorphic morphology and swelling of cardiac mitochondria compared to the control group. All of the pharmacological interventions could restore cardiac mitochondrial morphology which was obtained by using a transmission electron microscope in iron-overloaded rats.

ROS production was significantly increased in the HFe-fed rats when compared with the control group (Fig 5A). Although DFO, DFP, DFX and NAC treatments significantly reduced ROS production, only the combined DFP plus NAC treatment attenuated the ROS level to the same level as that observed in the control group (Fig 5A). Cardiac mitochondrial depolarization was observed as indicated by decreased red/green fluorescent intensity ratio in the HFe-fed rats when compared with the control group (Fig 5B). Although DFO, DFP, DFX and NAC monotherapy significantly reduced cardiac mitochondrial depolarization, only the combined DFP plus NAC led to the restoration of cardiac mitochondrial depolarization (Fig 5B). Consistently, cardiac mitochondrial swelling was significantly increased in the HFe-fed rats when compared with the control group (Fig 5C). All of the pharmacological interventions led to significant restoration of cardiac mitochondrial swelling in the iron-overloaded rats (Fig 5C). The representative electron micrographs demonstrated that the HFe-fed rats had unfolded cristae and irregularly shaped mitochondria, indicating mitochondrial swelling (Fig 6). Cardiac mitochondrial structure was effectively restored by all pharmacological interventions in the iron-overloaded rats (Fig 6).

## Discussion

The major findings of this study are as follows: 1) chronic iron loading for 4 months led to an iron overload condition developing in the rats, shown by an increase in the following: plasma NTBI and MDA, cardiac iron concentration, cardiac oxidative stress, cardiac mitochondrial dysfunction, cardiac sympathovagal imbalance and left ventricular dysfunction; 2) interventions by using DFO, DFP, DFX and NAC provided similar beneficial effects in reducing systemic and cardiac iron overload as well as lowering plasma and cardiac oxidative stress levels, leading to improved cardiac mitochondrial function, cardiac sympathovagal balance and left ventricular function; 3) combined DFP plus NAC treatment showed synergistic effects which were more marked than the results monotherapy gave in the heart tissue by attenuating and restoring cardiac iron concentration and cardiac MDA level, and restoring cardiac mitochondrial function, cardiac sympathovagal balance and left ventricular function; 4) this is the first study to demonstrate the protective effects of a combination of DFP and NAC treatment against iron overload induced-cardiac dysfunction and that this combination therapy significantly improved and restored cardiac function in iron-overloaded rats.

Under conditions of iron overload, plasma NTBI or free iron accumulation is caused by saturated-transferrin bound iron [6, 7]. Increased plasma NTBI leads to an increase in the entry of free iron into heart tissues through both LTCCs [9, 10] and TTCCs [11, 12]. Excess free iron in cardiac cells causes increased oxidative stress via Haber-Weiss and Fenton’s reactions [13, 14]. In this study, plasma NTBI and cardiac iron concentrations in the HFe-fed group were increased, indicating an iron overload condition in this animal model. Iron overload causes the production of ROS leading to the lipid-peroxidation of cellular membranes as well as damage to cellular proteins, nucleic acids and organelles [13, 14]. MDA is the end product of lipid peroxidation and is used as an indicator of oxidative stress and cell injury [54, 55]. This study showed that MDA levels were increased in both the plasma and the heart tissues of the rats with iron-overload, thus demonstrating an increase in systemic and intracardiac oxidative stress levels. In addition, cardiac mitochondria were also damaged by increased oxidative stress and mitochondrial depolarization, also mitochondrial swelling occurred in the iron-overloaded rats. Since mitochondria are vital organelles and function as the power houses of cells [56], iron-mediated oxidative stress caused cardiac mitochondrial damage and dysfunction, leading to impaired LV function as observed in this study. Moreover, since oxidative stress has a strong influence on the changes in the HRV [4, 11, 57], increased oxidative stress in iron overloaded rats could lead to impaired HRV as shown in this study. These mechanisms could help explain and support the findings that iron-overloaded rats had impaired LV function as shown by decreased %LVEF together with decreased LVESP, Pmax, cardiac output and stroke volume. This impairment was similar to that observed in TDT patients who often exhibit LV dysfunction [58, 59]. In the present study, although DFO, DFP, DFX or NAC treatment could improve LVESP, Pmax, cardiac output and stroke volume in the HFe-fed rats, only a combination of DFP plus NAC facilitated the restoration of LVESP, Pmax and cardiac output to a normal level, thus demonstrating that the combined therapy exerted better protection on cardiac mitochondrial function than monotherapy, leading to restoration of the LV function in iron-overloaded rats.

Currently, iron chelation is the main therapy used to treat patients with conditions of iron overload. DFO, DFP and DFX (three common iron chelators) have been shown to attenuate myocardial iron deposition, which led to improved cardiac function in TDT patients [26–29, 32, 33]. A previous study showed that NAC (a potent antioxidant) [34] has the ability to diminish oxidative stress and DNA damage due to iron-induced toxicity in β-thalassemia patients [36]. This current study demonstrated that despite plasma NTBI and MDA levels being attenuated by all treatments, these two parameters did not reach the normal levels as shown by the results from the control group. This result suggests that all of the pharmacological interventions had similar efficacy in reducing systemic NTBI and MDA levels. In reference to cardiac iron overload, the present study found that DFO, DFP, DFX and NAC demonstrated protective effects on cardiac dysfunction by reducing cardiac iron deposition, lowering oxidative stress, and improving cardiac mitochondrial function as well as improving the HRV and LV function in iron-overloaded rats. However, their protective effects did not sufficiently restore the LV function to reach the normal levels shown in the normal diet/control group. Although the present study and other [60] demonstrated that two oral iron chelators DFP and DFX had a similar efficacy in reducing cardiac iron and improving cardiac function in iron-overloaded rats, inconsistent findings exist regarding the effects of DFP and DFX. It has been reported that DFP was more effective than DFX in reducing cardiac iron and improving systolic ventricular function in TDT patients [37, 38]. In addition, DFX was shown to fail to remove cardiac iron in TDT patients with severe liver iron store [33]. Nevertheless, a large clinical study found that DFX was more effective than DFP in reducing liver iron content in TDT patients [38]. Additionally, although NAC did not cause any reduction in cardiac iron concentration to a normal level, it could decrease cardiac MDA content in the HFe-fed rats to a normal level (that found in the control group). This benefit might result from its anti-oxidative effects [34, 36].

Interestingly, a previous study showed that a combination of DFP plus NAC therapy exerted greater efficacy than monotherapy in attenuating brain oxidative stress and restoring brain function impaired by conditions of iron overload found in iron-overloaded rats [18]. In the present study, it was demonstrated that a combination of DFP plus NAC provides better cardioprotective effects than a monotherapy and could effectively reduce cardiac iron concentration and oxidative stress as well as being able to diminish mitochondrial ROS levels, mitochondrial depolarization and swelling to the extent of reaching normal levels as shown by the control group. Finally, cardiac sympathovagal balance was also effectively restored by the combined therapy, followed by improved and restored LV function in iron-overloaded rats.

**Fig 7:**
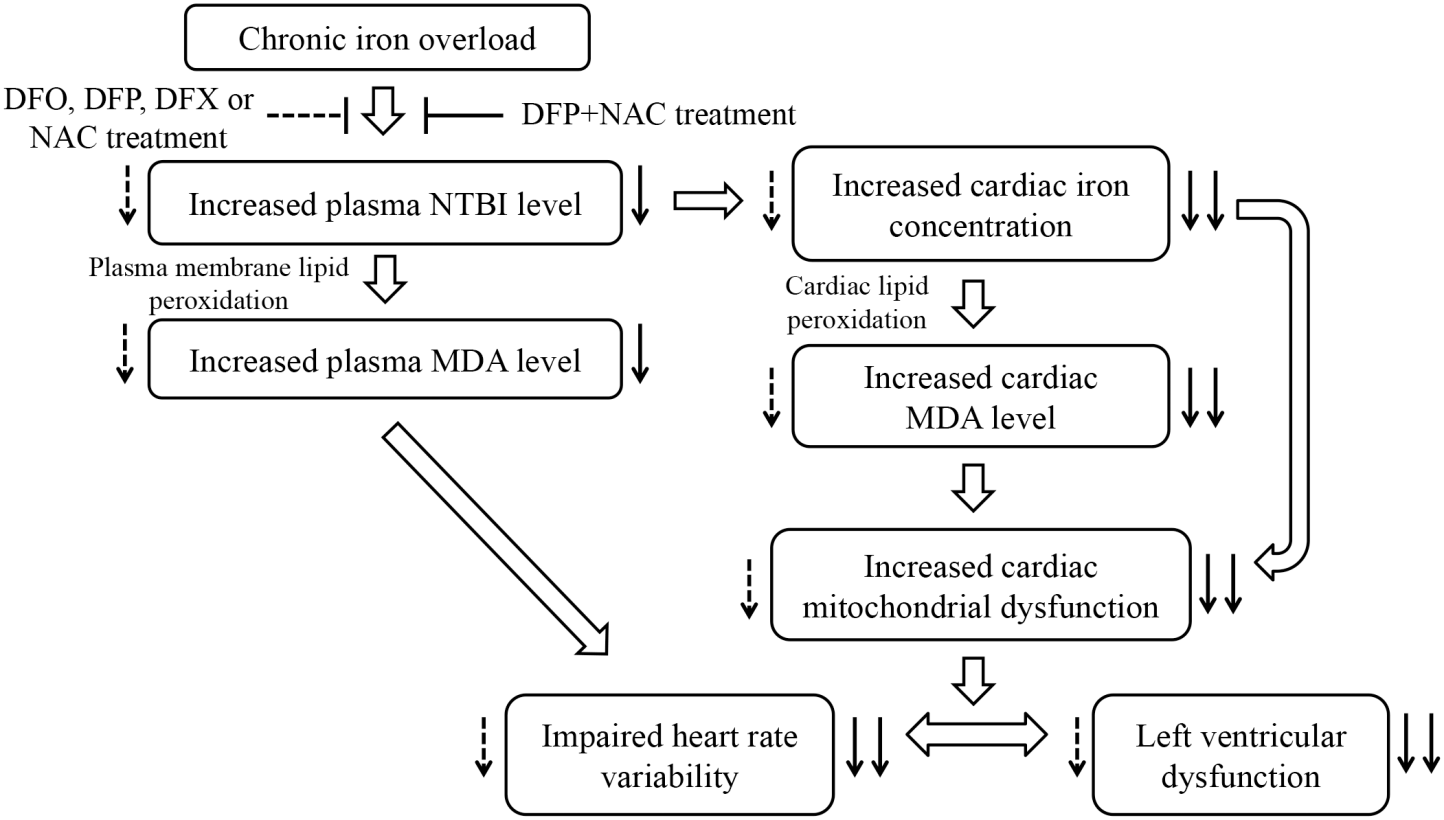
Diagram illustrating the proposed mechanisms of chronic iron overload on cardiac dysfunction and the effects of all pharmacological interventions on cardiac dysfunctions caused by chronic iron overload. Dashed arrows indicate the effects of either DFO, DFP, DFX or NAC treatment; solid arrows indicate the effects of combined DFP plus NAC treatment. DFO = deferoxamine; DFP = deferiprone; DFX = deferasirox; NAC = N-acetyl cysteine; NTBI = non-transferrin bound iron; MDA = malondialdehyde.

The combination of an iron chelator and an antioxidant may provide these effectively protective effects on iron overload-induced cardiac dysfunction by several mechanisms. Firstly, DFP can chelate free iron in the cell [61] which leads to decreased cardiac iron concentration, and therefore results in a decrease in oxidative stress caused by Haber-Weiss and Fenton’s reactions. Secondly, NAC can scavenge free radicals [34] and it is also known to have metal-chelating properties [62] including iron [63] which leads to decreased levels of both ROS and cardiac iron concentration. NAC might also complex with NTBI in the plasma and excreted by the kidney via glomerular infiltration [63]. Therefore, in this study a combination of DFP plus NAC provided synergistic cardioprotective effects and thus significantly improved and restored cardiac function in iron-overloaded rats. The mechanisms of chronic iron overload on cardiac dysfunction and the effects of all pharmacological interventions on cardiac dysfunction in iron-overloaded rats are summarized in Fig 7. These findings identify the need for future clinical trials to determine and warrant the clinical usefulness of combined iron chelator and NAC therapy in patients with iron overload cardiomyopathy.

## Conclusions

DFO, DFP, DFX and NAC monotherapy had similar efficacy in improving cardiac function in iron-overloaded rats. Interestingly, combined DFP plus NAC had synergistically therapeutic benefits and exerted more robust beneficial results than monotherapy in its cardioprotective effects. This was shown by restored cardiac iron concentration, oxidative stress, and cardiac mitochondrial function which led to restored cardiac sympathovagal balance and LV function to a normal physiological condition in iron-overloaded rats. This study may provide extensive insights into future therapeutic strategies for better treatment of iron overload cardiomyopathy.

## Limitations

A previous study indicated that DFO and BHT were added into the homogenized buffer to prevent iron-catalyzed, *ex vivo* MDA formation for determining MDA content in tissues [64]. Since DFO was not added in this study, it might be possible that the exogenous MDA was included in the measured MDA level. Also, MDA was used for oxidative stress status in the present study. Previous studies reported that 8-isoprostane is a prostaglandin (PG)-F_2_-like compound belonging to the F_2_ isoprostane class which is produced by the free radical-catalyzed peroxidation of arachidonic acid [65, 66]. 8-isoprostane is an accurate marker of lipid peroxidation and has been used to determine oxidative stress in *in vivo* [66, 67], especially in iron-overloaded rat model [68], which could be more specific to oxidative stress than MDA.
